# Genome-Wide Characterization of the Mitogen-Activated Protein Kinase Gene Family and Their Expression Patterns in Response to Drought and Colletotrichum Gloeosporioides in Walnut (*Juglans regia*)

**DOI:** 10.3390/plants12030586

**Published:** 2023-01-29

**Authors:** Kaiyu Yang, Jianghao Wu, Xialei Wang, Han Li, Peng Jia, Haoan Luan, Xuemei Zhang, Suping Guo, Minsheng Yang, Qinglong Dong, Guohui Qi

**Affiliations:** 1 College of Forestry, Hebei Agricultural University, Baoding 071001, China; 2 Technology Innovation Center of Hebei Province, Xingtai 054000, China; 3 Institute of Walnut Industry Technology of Hebei Province (Xingtai), Lincheng 054300, China

**Keywords:** MAPK gene family, *Juglans regia*, genome-wide analysis, *Colletotrichum gloeosporioides*, drought stress, expression analysis

## Abstract

Mitogen-activated protein kinases (MAPKs) are a family of Ser/Thr (serine/threonine) protein kinases that play very important roles in plant responses to biotic and abiotic stressors. However, the MAPK gene family in the important crop walnut (*Juglans regia* L.) has been less well studied compared with other species. We discovered 25 *JrMAPK* members in the *Juglans* genome in this study. The *JrMAPK* gene family was separated into four subfamilies based on phylogenetic analysis, and members of the same subgroup had similar motifs and exons/introns. A variety of cis-acting elements, mainly related to the light response, growth and development, stress response, and hormone responses, were detected in the *JrMAPK* gene promoters. Collinearity analysis showed that purification selection was the main driving force in *JrMAPK* gene evolution, and segmental and tandem duplications played key roles in the expansion of the *JrMAPK* gene family. The RNA-Seq (RNA Sequencing) results indicated that many of the *JrMAPK* genes were expressed in response to different levels of *Colletotrichum gloeosporioides* infection. *JrMAPK1*, *JrMAPK3*, *JrMAPK4*, *JrMAPK5*, *JrMAPK6*, *JrMAPK7*, *JrMAPK9*, *JrMAPK11*, *JrMAPK12*, *JrMAPK13*, *JrMAPK17*, *JrMAPK19*, *JrMAPK20*, and *JrMAPK21* were upregulated at the transcriptional level in response to the drought stress treatment. The results of this study will help in further investigations of the evolutionary history and biological functions of the MAPK gene family in walnut.

## 1. Introduction

Biotic and abiotic stressors occur frequently during plant growth and development, in which drought stress and infection by pathogens seriously affect crop yield and quality [1]. Mitogen-activated protein kinases (MAPKs) are Ser/Thr (serine/threonine) protein kinases and important components of the MAPK cascade signaling pathway. MAPKs are phosphorylated by MAPKK during the signal transduction process [2]. They directly act on target proteins and cause a cascade of physiological and biochemical processes in cells. MAPKs play an important role in regulating cell division, plant growth, and development [3,4].

The Arabidopsis MAPK gene family can be divided into TEY and TDY subtypes based on the characteristics of the conserved domains. The common feature of the TEY subtype of MAPK members is that they contain the T-E-Y (Thr-Glu-Tyr) motif, which can be further divided into groups A, B, and C. The TDY subtype is also called group D, which is characterized by the T-D-Y (Thr-Asp-Tyr) domain [4]. The roles of the Arabidopsis MAPK gene family members *AtMPK3*, *AtMPK4*, and *AtMPK6* have been extensively researched. For instance, the transcription levels of *AtMPK3*, *AtMPK4*, and *AtMPK6* are induced by osmotic stress, salt, cold stress, and a variety of pathogens [5,6,7]. Mutant and overexpressing transgenic plants have revealed that *AtMPK3*, *AtMPK4*, and *AtMPK6* play critical roles in both abiotic and biotic stress reactions [5,6,7]. *AtMPK3* and *AtMPK6* also play important roles in stomatal development, embryonic development, anther and ovule development, and inflorescence formation [8,9,10,11].

Several MAPK transcription factors of diverse species have been excavated and identified with the release of the genomic databases of various species, enabling researchers to have a deep understanding of the MAPK family members [12,13,14,15,16,17]. However, recognition and understanding of the walnut MAPK family members remain limited. As an edible nut, walnut (*Juglans regia* L.) has rich nutritional value and is popular with people all over the world. It is widely planted in temperate regions and is one of the most widely distributed, commercially important tree species. The genomic database of the ‘Chandler’ cultivar has been sequenced, providing a good platform for mining and identifying walnut MAPK family members at the genomic level [18,19]. In this work, we mined and identified 25 members of the MAPK gene family in the walnut genome and analyzed their evolutionary relationships, chromosome locations, conserved elements, and intron/exon structures, as well as investigated the expression levels of *JrMAPK* genes under drought stress and *Colletotrichum gloeosporioides* infection. The results will provide new insight and the basis for further investigation on the biological functions of MAPK gene family members in walnut.

## 2. Results

### 2.1. Identification and Physiological Properties of the MAPK Genes

A total of 25 *JrMAPK* genes were identified from the walnut genomic database and named *JrMAPK1*–*JrMAPK25* (Table S3), according to their position on the chromosome. The identified *JrMAPK* genes were 179 (*JrMAPK16*) to 623 (*JrMAPK2*) amino acids in length. The molecular weights were from 37.52 (*JrMAPK24*) to 70.76 (*JrMAPK2*) kDa. The isoelectric points were from 4.79 (*JrMAPK16*) to 9.63 (*JrMAPK1*). The predicted total average hydrophilicity (GRAVY) score ranged from −0.579 (*JrMAPK22*) to −0.07 (*JrMAPK16*). The instability index ranged from 33.38 (*JrMAPK14*) to 46.83 (*JrMAPK5*), and 14 *JrMAPK* proteins were unstable. According to subcellular localization prediction, 25 JrMAPK proteins were found in the nucleus. In addition, none of the *JrMAPK* genes had signal peptides (Table S3).

### 2.2. Multiple Sequence Alignment and Phylogenetic Analyses of the MAPK Genes

**Figure 1:**
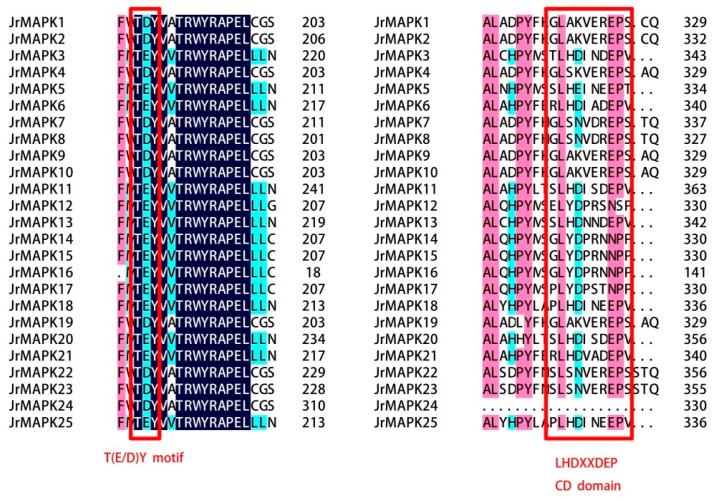
Multiple sequence alignment of proteins in the Mitogen-activated protein kinase (MAPK) gene family in *Juglans regia*. Dark blue highlighted residues are identical, while other colored highlighted residues are similar in all proteins. Red boxes indicate the TEY/TDY motifs and the conserved docking (CD) domain.

**Figure 2:**
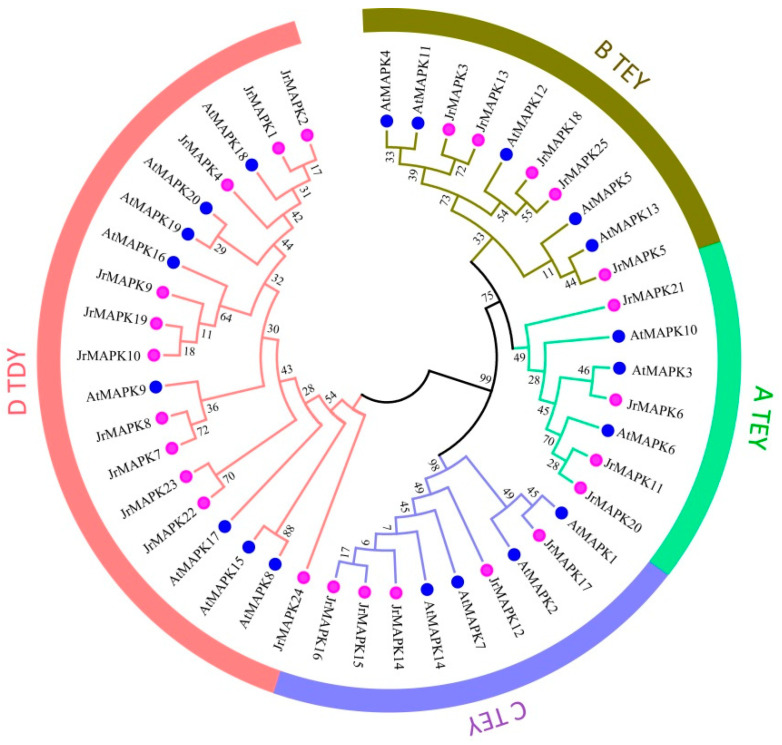
Evolutionary analysis and group classification of the *AtMAPK* (*Arabidopsis thaliana*) and *JrMAPK* (*Juglans regia*) proteins. The neighbor-joining (NJ) method in MEGA5.0 was used to construct the phylogenetic tree. Bootstrap values from 1000 replicates are displayed at each node. A–D indicate the different groups of MAPKs.

After comparing the amino acid sequences of the 25 *JrMAPK* proteins, all walnut MAPK family members contained TXY, a highly conserved tripeptide motif (Figure 1), in which *JrMAPK3*, *JrMAPK5*, *JrMAPK6*, *JrMAPK11*, *JrMAPK12*, *JrMAPK13*, *JrMAPK14*, *JrMAPK15*, *JrMAPK16*, *JrMAPK17*, *JrMAPK18*, *JrMAPK20*, *JrMAPK21*, and *JrMAPK25* contained TEY, and the other members contained TDY. Similarly, the conserved docking site LHEDXXDEP (Figure 1) was also observed in most of the *JrMAPKs*. A phylogeny tree was created from the protein sequences of the 25 *JrMAPK* genes from walnut and the 20 *AtMAPK* genes from Arabidopsis to study the evolution connections among the *JrMAPK* proteins (Figure 2). The results showed that the 25 *JrMAPKs* were divided into groups A, B, C, and D. The TEY motif is found in members from groups A, B, and C, while the TDY motif is found in members from group D. *JrMAPK6*, *JrMAPK11*, *JrMAPK20,* and *JrMAPK21* were assigned to subgroup A; *JrMAPK3*, *JrMAPK5*, *JrMAPK13*, *JrMAPK18*, and *JrMAPK25* were assigned to subgroup B; *JrMAPK12*, *JrMAPK14*, *JrMAPK15*, *JrMAPK16*, and *JrMAPK17* were assigned to subgroup C; and *JrMAPK1*, *JrMAPK2*, *JrMAPK4*, *JrMAPK7*, *JrMAPK8*, *JrMAPK9*, *JrMAPK10*, *JrMAPK19*, *JrMAPK22*, *JrMAPK23*, and *JrMAPK24* were assigned to subgroup D (Figure 2).

### 2.3. Gene Structure and Conserved Motif Analysis

**Figure 3:**
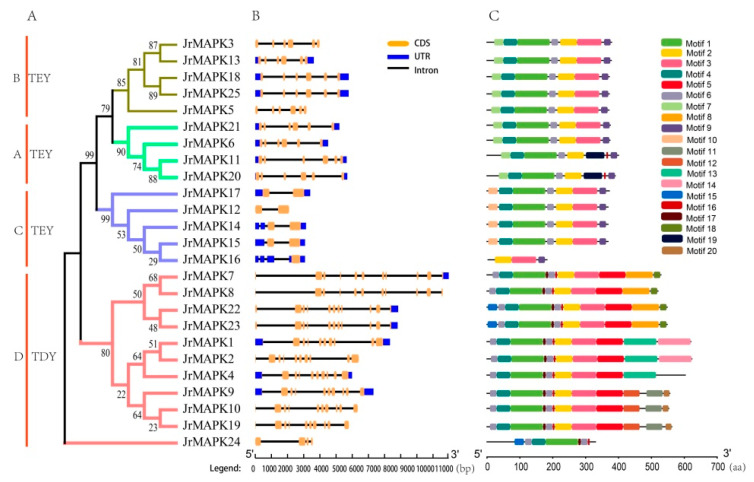
Structure and conservative motif analysis of the *JrMAPK* genes. (**A**) Phylogentic analysis of MAPK proteins in Juglans. (**B**) The gene structure of MAPK family in Juglans. (**C**) The distribution of conserved motifs in *JrMAPK* proteins. Each motif is represented by a colored box.

The motif analysis revealed that 20 motifs were identified in the 25 *JrMAPK* protein sequences, and MAPK genes with closer homology usually contained the same motif composition and sequence arrangement (Figure 3C). For example, all members of group B contained motifs 7, 4, 1, 6, 2, 3, and 9. In addition, motifs 6, 4, and 1 occurred in all *JrMAPK* genes, except *JrMAPK16* in group C; motifs 2 and 3 existed in all *JrMAPK* genes, except *JrMAPK24* in group D. Some conserved motifs only existed in specific groups. For instance, motif 10 only existed in group C, motif 19 only existed in group A, and motifs 5, 8, 11, 12, 13, 14, 15, 17, 18, and 20 only existed in group D.

We also analyzed the gene structure of the *JrMAPKs* using GSDS (Gene Structure Display Server) to reveal the exon/intron arrangement of each gene (Figure 3B). The distribution, number, and length of exons/introns vary from gene to gene. The number of introns varied from 1 (*JrMAPK12*, *JrMAPK17*) to 10 (*JrMAPK7*, *JrMAPK8*, *JrMAPK22*, and *JrMAPK23*) (Figure 3B), which is similar to the results of tomato [20] and cucumber [21]. All of the *JrMAPK* genes in groups A and B had five introns. Group C genes contained 1–3 introns, and group D genes contained 3–10 introns. These results show that the structure of the *JrMAPK* genes in groups A and B was more conserved than that in groups C and D, based on the complex distribution of the exons and introns. In addition, most of the genes in the same subgroup had similar exon/intron structures. For example, two genes in group A (*JrMAPK18* and *JrMAPK25*) and two genes in group D (*JrMAPK22* and *JrMAPK23*) had the same intron-exon distribution pattern.

### 2.4. Chromosome Localization and Gene Replication in the MAPK Proteins

**Figure 4:**
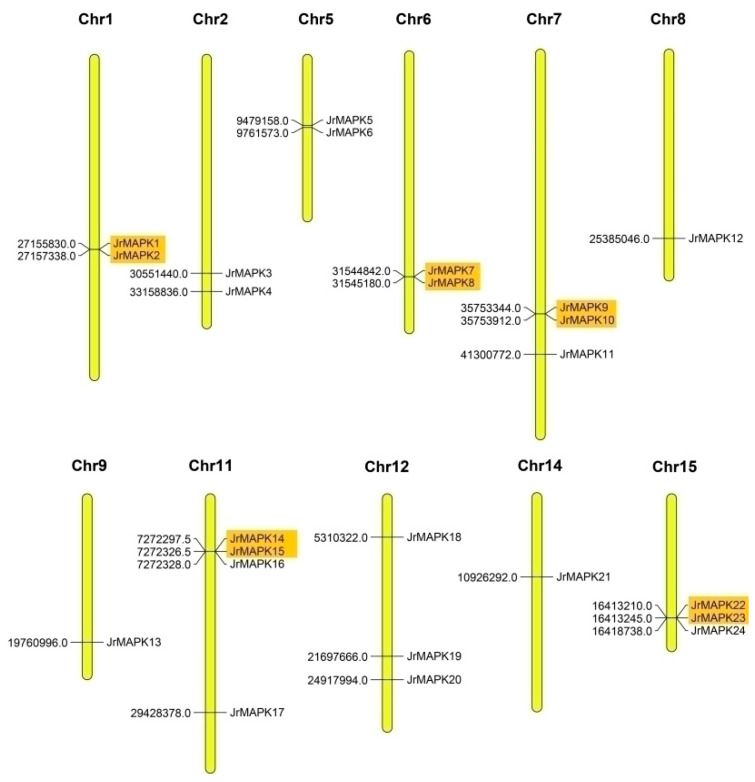
Chromosomal mapping of the *JrMAPK* genes. The vertical columns represent chromosomes. Chromosome names show on the top of vertical columns. Gene names shown on the right of vertical columns. Gene location shown on the left of vertical columns. Orange boxes indicate tandem duplications.

**Figure 5:**
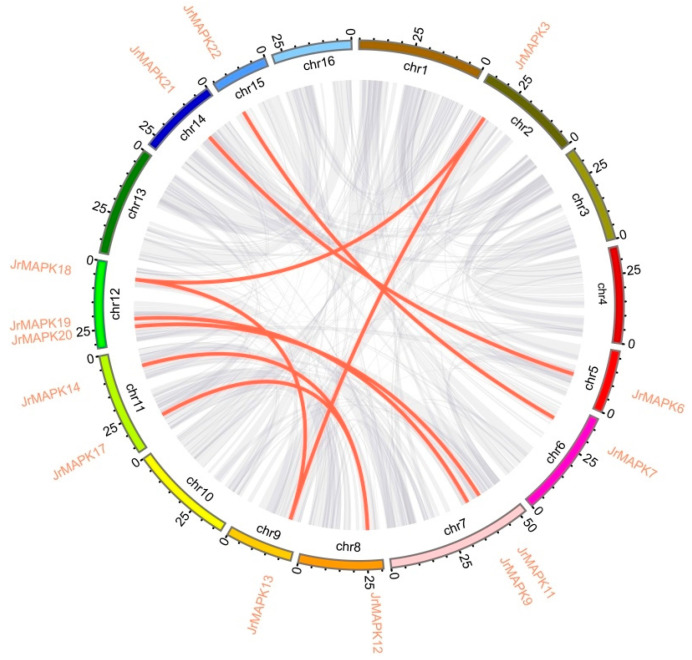
The synteny of MAPK genes in Juglans. Red lines represent segmental duplications of MAPK gene pairs, while gray lines represent synteny blocks in the walnut genome. Each chromosome has an indication of its chromosome number at the bottom.

We analyzed the distribution of the *JrMAPK* genes on the chromosomes. As shown in Figure 4, except for one gene (*JrMAPK25*) located on the scaffold, 24 *JrMAPK* genes were unevenly distributed on 11 chromosomes. The number of *JrMAPK* genes on chromosome 11 was the largest, with 4 *JrMAPK* genes. Next were Chr7, Chr12, and Chr15, which had five *JrMAPK* genes each. Chr1, Chr2, Chr5, and Chr6 each had two *JrMAPK* genes. On chromosomes 8, 9, and 14, only one *JrMAPK* gene was discovered each. We studied gene duplication events to elucidate the potential expansion mechanism of the *JrMAPK* gene family, including tandem duplication and segmental duplication (Figure 5 and Table S4). The results showed that various *JrMAPK* genes (80%, 20/25) existed as 2 or more copies. A total of 10 *JrMAPK* genes underwent tandem replication, and 14 *JrMAPK* genes underwent segmental replication. In order to determine the driving force of the evolution of the *JrMAPK* gene family, we also studied the nonsynonymous and synonymous substitution ratio (Ka and Ks) of duplicate genes (Table S4). The results showed that the Ka/Ks ratios of 10 pairs of genes were <1, demonstrating that purifying selection played a major role in the evolution of the *JrMAPK* genes.

### 2.5. Analysis of Cis-Acting Elements in the JrMAPK Promoters

**Figure 6:**
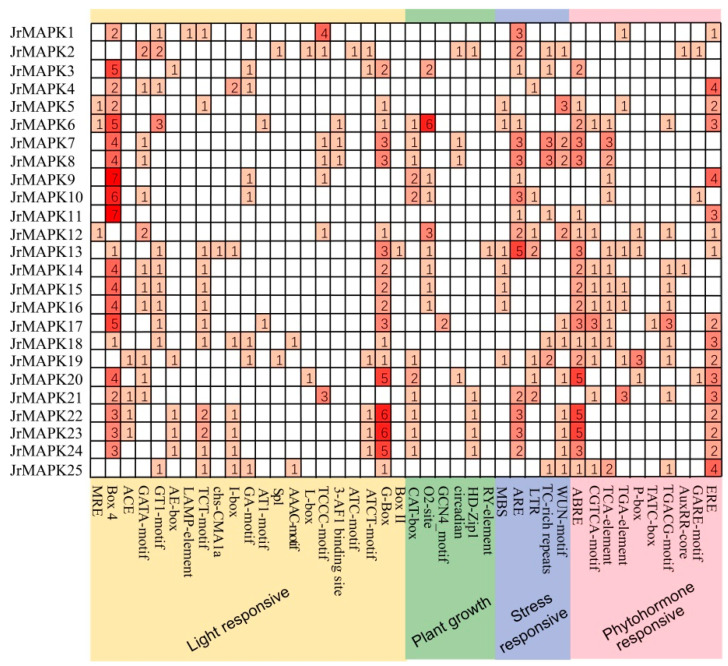
Distribution of the cis-acting elements in the promoters of the *JrMAPKs*.

We investigated the cis-acting elements in the *JrMAPK* promoter region further. The *JrMAPK* promoter cis-acting elements were classified into four groups: light response, stress response, plant growth and development, and plant hormone response-related elements (Figure 6). The G-box, box 4, GATA motif, and GT1 motif were among the light response elements. This finding indicated that *JrMAPK* genes may play a role in light response-mediated regulating. *JrMAPK* genes were found to be involved in the regulation of hormone signaling pathways, including gibberellin (GA), abscisic acid (ABA), and other hormone response elements. Additionally, the discovery of stress-response-related elements, such as MBS (MYB binding site implicated in the stress response), ARE (anaerobic induction of cis-elements), and the LTR (low-temperature response element), showed that *JrMAPK* genes are engaged in the stress response. We also detected CAT box, circular elements, and RY elements, etc., which are cis-acting elements associated with growth and development.

### 2.6. Expression Profiles of JrMAPK Genes in Response to Drought Stress and Fungal Infection

**Figure 7:**
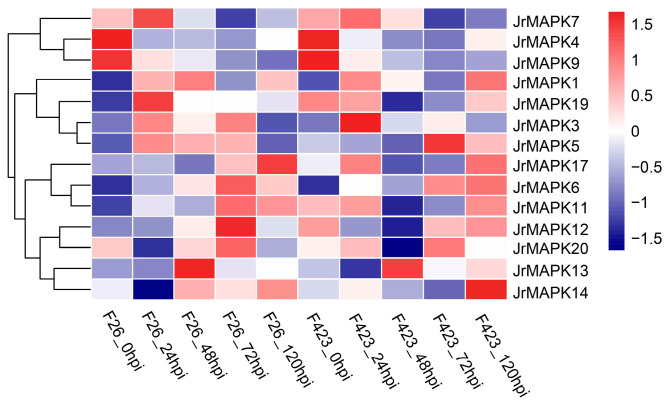
Transcriptional abundance of the *JrMAPK* genes in *Colletotrichum gloeosporioides* infections. Colored bars indicate normalized FPKM values, with red indicating high levels of expression, white no expression, and dark blue low expression. The detailed FPKM values are shown in Table S1.

To explore the function of the *JrMAPK* genes in the defense reaction of walnuts, we analyzed the transcriptional regulation of 14 *JrMAPK* proteins after infection with *C. gloeosporioides* (Figure 7). According to the outcomes, the transcription levels of *JrMAPK* proteins in F26 (anthracnose-resistant) and F423 (anthracnose-susceptible)were induced by *C. gloeosporioides* infection. Among them, the expression levels of *JrMAPK3* in F26 and F423 were 4.25 and 3.14 times higher, respectively, at 72 hpi (hours post inoculation) than those at 0 hpi, and the transcription levels of *JrMAPK6* in F26 and F423 were 2.20 and 1.57 times higher, respectively, than those at 0 hpi. The transcription levels of *JrMAPK1* and *JrMAPK13* in F26 were 1.94 and 1.93 times higher at 48 hpi than those at 0 hpi, and 1.17 and 1.45 times higher in F423 than at 0 hpi. Moreover, the transcription levels of *JrMAPK4*, *JrMAPK7*, and *JrMAPK9* were downregulated by *C. gloeosporioides* infection in F26 and F423. *JrMAPK5* expression was upregulated in F423 and downregulated in F426. The expression levels of *JrMAPK11*, *JrMAPK12*, and *JrMAPK19* were downregulated in F423 and upregulated in F26. The transcription pattern of the other JrMAPK proteins changed not significantly.

**Figure 8:**
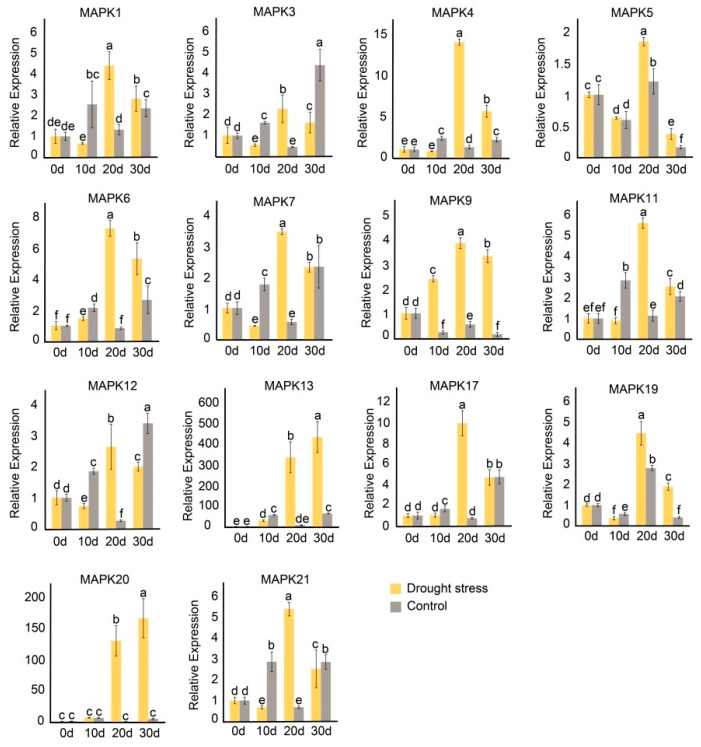
Expression pattern of the *JrMAPK* genes under drought treatment. Error bars are standard deviations from the biologic replicates. Bars with different letters in each panel are significantly different at *p* < 0.05, based on one-way ANOVA and Tukey’s test.

Next, the expression profile of *JrMAPKs* under drought stress was detected by Quantitative Real-time Polymerase Chain Reaction (qRT-PCR). As indicate in Figure 8 and Table S5, compared with the control, the expression patterns of most of the *JrMAPK* genes were significantly up-regulated after drought stress and were many times higher than that of the control, but the peak times varied between the genes. Among them, *JrMAPK1*, *JrMAPK3*, *JrMAPK4*, *JrMAPK5*, *JrMAPK6*, *JrMAPK7*, *JrMAPK9*, *JrMAPK11*, *JrMAPK12*, *JrMAPK17*, *JrMAPK19*, and *JrMAPK21* reached their highest expression levels 20 days after the drought stress, and their gene expression levels were 3.33, 5.07, 10.89, 1.53, 8.55, 6.21, 6.87, 4.93, 10.01, 13.21, 1.60, and 7.94 times higher, respectively, than those of the control. In contrast, the expression of *JrMAPK13* and *JRMAPK20* were highest 30 days after the drought stress, and their gene expression levels were 6.80 and 34.57 times higher, respectively, than those of the control.

### 2.7. Protein–Protein Interaction of JrMAPK Protein

**Figure 9:**
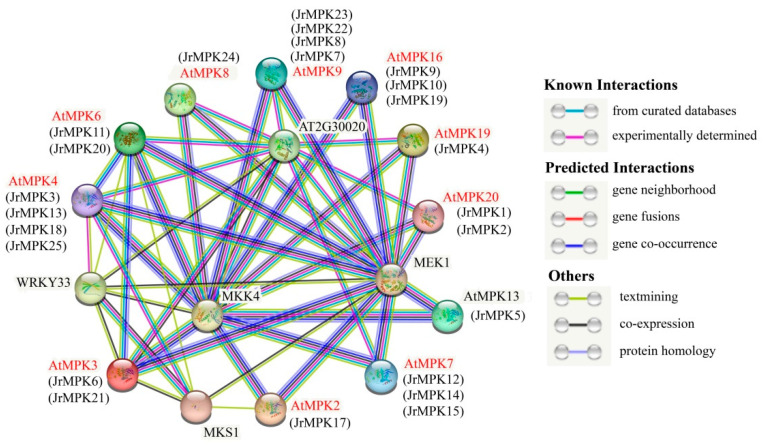
MAPK protein interaction network diagram. Proteins are represented by the network nodes. The 3D structure of the protein is displayed inside the node, and the line color indicates the type of interaction evidence.

In order to analyze the function of JrMAPK proteins, the interaction network map was constructed based on the homologous protein family in Arabidopsis. Using well-studied *AtMAPK* genes, we predicted several important interactions and preliminarily speculated the possible functions of some *JrMAPK* genes (Figure 9 and Table S6). Interestingly, there is obvious homology between *AtMAPK4* (homologue of *JrMAPK3/13/18/25*) and *MKS1* genes. *MKS1* encodes nuclear localized members of the plant-specific gene family and participates in mediating the response to pathogens. *MEK1* and *AtMAPK4/6* plays a role in a signal pathway that regulates gene expression in response to biotic and abiotic stresses and plays an important role in pathogen defense through negative regulation of innate immunity. The *MKK1*-*AtMPK6* module responds to drought and salt stress by mediating ABA-dependent *CAT1* expression. During seed germination, the *MKK1*-*AtMPK6* module is also implicated in sugar signaling. *MPK3* and *MPK6* activate upstream *MKK4* by phosphorylation. Stomatal cell fate is regulated by the *MKK4-MPK3*/*MPK6* module before the guard mother cell (GMC) is determined. In addition, *AtMAPK3* (homologue of *JrMPK6/21*), *AtMAPK4* (*JrMAPK3/13*), and *AtMAPK6* (homologue of *JrMAPK11/20*) are predicted to interact with *WRKY33*, and *WRKY33* is involved in plant defense response to fungal pathogens. *AtMAPK4* (homologue of *JrMPK3/13/18/25*) and *AtMAPK6* (homologue of *JrMAPK11/20*) interact with *AT2G30020*. *AT2G30020* is a protein phosphatase that negatively regulates the defense response. Inactivated *MPK4* and *MPK6* MAP kinases are involved in stress and defense signals.

## 3. Discussion

Walnut trees are often exposed to abiotic and biotic stressors during growth and development. In particular, the *C. gloeosporioides* fungus has had a tremendous impact on the growth and development of walnut and fruit quality [22]. Various studies have shown that the MAPK transcription factors play key roles in the reaction to abiotic and biotic stressors [23]. Therefore, we explored and identified members of the walnut MAPK gene family, as well as their expression patterns in response to abiotic and biotic stressors. The MAPK gene families have been identified in *Solanum lycopersicum* [20], maize [24], apple [25], tobacco [26], Arabidopsis [27], and potato [28], with 16, 19, 26, 17, 20, and 22 members, respectively. In this investigation, 25 *JrMAPK* genes were discovered in the Juglans genome.

It is now generally believed that the MAPK gene family can be categorized into groups A, B, C, and D [13,29,30]. There are 12 members in the ABC subgroups of Arabidopsis, which contained the conservative TEY phosphorylation domain at its phosphorylation site. Subgroup D has eight members, and there is a TDY phosphorylation motif in the corresponding position [31]. In this work, the *JrMAPK* transcription factors were divided into groups A, B, and C, containing the TEY motif, and group D, containing the TDY motif. In addition, all MAPK members had a CD domain, except for *JrMAPK24* in group D, which has been described as LHDXXDEP. This is consistent with the descriptions for *Brassica napus* and *Solanum tuberosum*. The members of groups C and D also have CD domains in these two species [28,32].

The positions of the exons and introns in the genome generally offer crucial evidence for phylogenetic analysis. The structural distribution and length of exons and introns of *JrMAPK* gene family members were systematically and comprehensively analyzed in this study. The *JrMAPK* gene family members were composed of 1–10 exons. Groups A and B had 5 introns, Group C contained 1–3 introns, and Group D contained 3–10 introns. The *JrMAPK* genes in the same subgroup had comparable intron numbers and exon lengths. Similar structural patterns have been observed in other plants, which are highly conserved within the group, but large differences are observed between groups [33,34]. Our analysis of the conserved motif domains revealed that most members of the same subset had comparable conserved motifs, and the differences between different subgroups are large, which means that the protein structure of a particular subgroup is conserved. All *JrMAPK* genes contained motif 4, except *JrMAPK16*, which is different from other species [17,30,35]. It may be that *JrMAPK16* lacks some amino acids before TEY in motif 4. The reliability of our subfamily classification is strongly supported by the similar conserved motif and gene structure analysis of MAPK genes in the same branch.

Various studies have shown that gene replication (including tandem replication and segmental replication) is crucial for genome expansion and rearrangement, as well as in in the diversification of gene function and the generation of a large number of gene families [36]. In this study, the chromosome mapping investigation discovered that JrMAPKs were irregularly distributed in 11 of the 16 chromosomes. At least 10 *JrMAPK* genes underwent tandem duplication (Figure 4), and at least 14 *JrMAPK* genes underwent segmental duplication (Figure 5). These findings suggest that gene replication is a critical factor in the expansion of the walnut MAPK gene family. The Ka/Ks values of the 10 pairs of duplicate genes were <1, indicating that these *JrMAPKs* may have experienced purifying selection. The ratio of *JrMAPK7*/*JrMAPK8*, *JrMAPK9*/*JrMAPK10*, and *JrMAPK22*/*JrMAPK23* was “NA”, which could be attributed to the high level of differentiation in the genetic sequence, resulting in a long evolutionary distance [37]. As a result, the research shows that the Ka/Ks values of almost all of the *JrMAPK* proteins were <1, which has been reported in other gene families [38,39,40].

The analysis of cis-acting elements revealed that the *JrMAPK* gene promoter region contained a large number of cis-acting elements. For example, the existence of MYB elements, ABRE (ABA response elements), MYC elements, CGTCA motifs, TGACG motifs (metal jasmonate reaction motifs), and variable light response elements indicates that the *JrMAPK* genes may be involved in various stress signal transductions. Similar cis-acting elements of MAPK genes have been also identified in other species [21,35,41].

Many studies have shown that the plant MAPK cascade signaling pathway is widely implicated in various abiotic and biotic stress responses [42,43]. The most characteristic MAPKs in Arabidopsis are *AtMPK3*, *AtMPK4*, and *AtMPK6*, which are enabled by different triggers, such as pathogenic bacteria, abiotic stress, and oxidative stress [42]. In our study, the fold expression of *JrMPK6* and *JrMPK11,* which are homologous to *AtMPK3* and *AtMPK6* in anthracnose-resistant F26, was higher than that in anthracnose-susceptible F423 after being infected by *C. gloeosporioides*. *JrMPK3* and *JrMPK13*, which are homologous to *AtMPK4*, were also induced by *C. gloeosporioides*. This result confirms that the same group of MAPK proteins may have similar functions in different species [31,44]. In addition, *JrMPK1* and *JrMAPK12* in group C were upregulated in anthracnose-resistant F26, which is consistent with the results of research on group C in rice and alfalfa [45,46]. Protein–protein interaction network analysis shows that *JrMAPK3*, *JrMAPK6*, *JrMAPK11*, *JrMAPK13*, and *JrMAPK20* interact with *MKS1*, *MEK1*, and *WRKY33*, which are involved in mediating pathogen responses. We speculate that these genes have a significant role in walnut biological stress.

Drought stress is the main limiting factor in global crop yield [47]. Studies have shown that *AtMPK6* in subgroup A and *AtMPK4* in subgroup B participate in the abiotic stress reactions to cold and drought [6,48]. In the present study, 14 *JrMAPK* genes were significantly induced after drought stress. The gene expression levels of *JrMAPK20* in subgroup A and *JrMAPK13* in subgroup B were 34.57 and 6.80 times higher than those of their respective controls 30 days after the drought stress. In addition, some studies have reported that genes in groups C and D participate in the environmental stress response [49,50]. In this study, the gene expression levels of *JrMAPK12* and *JrMAPK17* in group C and of *JrMAPK4* in group D were more than 10 times higher than their respective controls 20 days after the drought stress. In the upstream regions, cis-acting elements relevant to the stress response were discovered, indicating that these genes are important in regulating drought conditions. Moreover, from the results of the interaction network, there is a close relationship between *JrMAPK3*, *JrMAPK6*, *JrMAPK11*, *JrMAPK13*, *JrMAPK20*, *JrMAPK21* and abiotic-related *MEK1*. This finding further emphasizes the specific function of these genes in regulating abiotic stress. In addition, most of the selected *JrMAPK* genes responded to two different stress conditions. For example, *JrMAPK1*, *JrMAPK3*, *JrMAPK5*, *JrMAPK6*, *JrMAPK11*, *JrMAPK12*, *JrMAPK13*, and *JrMAPK19* were not only induced by *C. gloeosporioides*, but also responded to drought stress, indicating that they were the main factors involved in crosstalk between different signal transduction pathways to cope with the response to the biotic and abiotic stressors. However, the specific biological functions of *JrMAPKs* under biological and abiotic stress need further experimental verification.

## 4. Materials and Methods

### 4.1. Identification of the JrMAPK Genes

The Ensembl database (http://plants.ensembl.org/index.html, accessed on 1 April 2022) was used to find the *Juglans regia* genome_v2.0 data. The serial/threshold protein kinase domain (PF00069) Hidden Markov Model file was obtained from the pfam database (http://pfam.xfam.org/, accessed on 1 April 2022). The walnut genome database was searched for MAPK genes using HMMER 3.0 (http://hmmer.org, accessed on 1 April 2022). The default settings were used, with a 0.01 cut-off value. To confirm that the predicted MAPK genes contained the conserved domains, they were submitted to the SMART (http://smart.embl.de/, accessed on 1 April 2022) and NCBI CDD (https://www.ncbi.nlm.nih.gov/cdd/, accessed on 1 April 2022) databases. The physical and chemical characteristics of the MAPK proteins, such as their isoelectric point, relative molecular weight, and instability index, were predicted using the online program ExPASy (https://web.expasy.org/comutepi/, accessed on 5 April 2022). The SignalP5.0 server (http://www.cbs.dtu.dk/services/SignalP/, accessed on 5 April 2022) was used to predict signal peptides. Subcellular localization was predicted using PlantmPLoc (http://www.csbio.sjtu.edu.cn/bioinf/plant-multi/, accessed on 5 April 2022).

### 4.2. Sequence Alignment and Phylogenetic Analysis

The 25 *JrMAPK* protein sequences underwent multiple sequence alignment using DNAMAN 6.0.3.99 (Lynnon Biosoft Corp., San Ramon, CA, USA) and its default settings. The full-length amino acid sequences of the MAPK proteins in Arabidopsis and walnut were utilized to create the evolutionary tree using the neighbor-joining (NJ) method and MEGA 5.0 software (www.megasoftware.net, accessed on 7 April 2022). The following parameters were used with the neighbor-joining (NJ) method: Complete deletion, amino, and bootstrap (1000 replications) p-distance [51].

### 4.3. Gene Structure, Motif Detection, and Cis-Acting Elements Analyses

We identified the conserved motifs of the *JrMAPK* gene family members using Meme v 5.0.2 (http://meme-suite.org/tools/meme, accessed on 10 April 2022) software [52]. The following optimal parameters were selected: the maximum number of motifs is 20, and the breadth of each motif is 6–100 residues. The exon/intron arrangement in the *JrMAPKs* gene structure was shown using the gene structure display server (GSDS) program (http://gsds.cbi.pku.edu.cn/, accessed on 10 April 2022) [53]. In order to find the potential cis-elements, the *JrMAPK* genes’ upstream 1.5 kb genomic sequences were uploaded to the PlantCARE database (http://bioinformatics.psb.ugent.be/webtools/plantcare/html/, accessed on 10 April 2022) [54]. Using 25 *JrMAPK* protein sequences as query conditions, homologous Arabidopsis genes were selected as reference. Protein interaction is predicted using String 10.0 (http://string-db.org/, accessed on 24 December 2020).

### 4.4. Chromosomal Location and Synteny Analysis

Based on the chromosomal positional information provided by the Ensembl database (http://plants.ensembl.org/index.html, accessed on 1 April 2022), chromosomal location images were obtained for the *JrMAPK* genes using Mapchart 2.32 software [55]. Duplicate gene pairs were searched using the BLAST method [5]. When the length of the aligned sequence covered more than 70% of the longer gene and the similarity of the aligned regions was greater than 70%, gene duplications were defined [56,57]. Tandem duplication was described as two or more genes placed on the same chromosome, one after the other, whereas segmentation duplication was defined as gene duplication on distinct chromosomes or within the same chromosome, but not one after the other [58]. Segmented duplicate genes were visualized with the Circos program [59]. KaKs Calculator 2.0 [60] was used to estimate the nonsynonymous substitution rate (Ka) and synonymous substitution rate (Ks) values and to calculate the Ka/Ks ratio.

### 4.5. Plant Materials and Treatments

Walnut seedlings that were 2 years old were raised in pots (diameter: 350 mm; height: 420 mm) in a greenhouse. The drought treatment was started when the plants reached a height of about 1500 mm. The designated control plants always received the normal irrigation plan, while the treatment group plants were suspended from irrigation for 30 days, and on days 0, 10, 20, and 30 of the water deficit, leaves were collected. The leaf tissues were instantly submerged in liquid nitrogen and kept at −80 °C.

The RNA-Seq (RNA Sequencing) data for the *JrMAPK* response to *C. gloeosporioides* were taken from Feng et al. [61] (Table S1). The test materials were fruit bracts of F26 (anthracnose-resistant) and F423 (anthracnose-susceptible). Both were inoculated with the *C. gloeosporioides* fungus, which had been grown on PDA media for 5–7 days in the dark at 28 °C. Samples were collected 0, 24, 48, and 72 hpi (hours post inoculation), and 0 hpi was used as the control.

### 4.6. RNA Extraction and Quantitative Real-Time-Polymerase Chain Reaction (qRT-PCR) Analysis

The Foregene plant total RNA isolation kit (LeiniaoBiolech, Chengdu, China) was used to extract total RNA. The cDNA was synthesized using the M5 sprint qPCR RT kit with the gDNA remover (Mei5 Biotechnology, Beijing, China), and primers were designed using Primer Premier 6.0 software (Palo Alto, CA, USA) (Table S2). Gene expression was carried out by RT-PCR, using the 2× SYBR Green qPCR Master Mix (BiolifeScience, Guangzhou, China) and the StepOnePlus Real-Time PCR System (Thermo Fisher Scientific, Wilmington, DE, USA), using GADPH as the internal control. Each reaction was performed in triplicate in a reaction volume of 20 μL. The qRT-PCR parameters were: 95 °C/5 s, 60 °C/15 s, and 72 °C/20 s, a total of 40 cycles. Each assay includes three biological replicates. The 2^−ΔΔCt^ method was used to estimate the fold gene expression. MeV4.8 tools (Boston, MA, USA) were applied to create a heatmap.

### 4.7. Statistical Analysis

SPSS Statistics 23 (IBM Corp., Armonk, NY, USA) was used to analyze all data, and the results of each treatment were compared using Duncan’s test and one-way analysis of variance. *p*-value < 0.05 was deemed significant.

## 5. Conclusions

In this study, we systematically analyzed the walnut MAPK gene family and identified 25 *JrMAPK* genes in the walnut genome. Based on bioinformatics methods, *JrMAPKs* were extensively analyzed for chemical and physical qualities, gene structure, conservative motifs, chromosomal location and evolution, and cis-acting elements. We discussed the response of the *JrMAPK* genes to drought stress and *C. gloeosporioides* infection. Fourteen selected genes were induced by drought stress. In addition, *JrMAPK1*, *JrMAPK3*, *JrMAPK5*, *JrMAPK6*, *JrMAPK11*, *JrMAPK12*, *JrMAPK13*, and *JrMAPK19* were highly expressed after *C. gloeosporioides* infection. Moreover, we constructed a protein interaction network, indicating that multiple MAPK genes are involved in the expression regulation of stress. These conclusions set a theoretical foundation for comprehending the stress resistance mechanism of walnut and establish a groundwork for future research on the evolution of the *JrMAPK* gene family and its function under biotic and abiotic stress.

## Data Availability

The data presented in this study are available in supplementary material.

## Supplementary Materials

The following supporting information can be downloaded at: https://www.mdpi.com/article/10.3390/plants12030586/s1, Table S1: RNA-Seq (RNA Sequencing) data on the response of *JrMAPK* to *Colletotrichum gloeosporioides*. Table S2: The specific primers for Quantitative Real-time Polymerase Chain Reaction (qRT-PCR). Table S3: The length distribution and physicochemical properties of *Juglans regia* MAPK proteins. Table S4: Segmentally and tandemly duplicated MAPK gene pairs in Juglans. Table S5. Expression profiles of *JrMAPK* genes under drought treatment. Table S6: Detail information about the predicted protein interaction network of JrMAPK proteins by STRING.
